# Designing Biological Microsensors with Chiral Nematic Liquid Crystal Droplets

**DOI:** 10.1021/acsami.2c06923

**Published:** 2022-08-15

**Authors:** Lawrence W. Honaker, Chang Chen, Floris M.H. Dautzenberg, Sylvia Brugman, Siddharth Deshpande

**Affiliations:** † Laboratory of Physical Chemistry and Soft Matter, Wageningen University & Research, Wageningen 6708 WE, The Netherlands; ‡ Host-Microbe Interactomics, Wageningen University & Research, Wageningen 6708 WD, The Netherlands

**Keywords:** liquid crystals, chiral nematic, biosensing, amphiphiles, zebrafish, on-chip detection assay

## Abstract

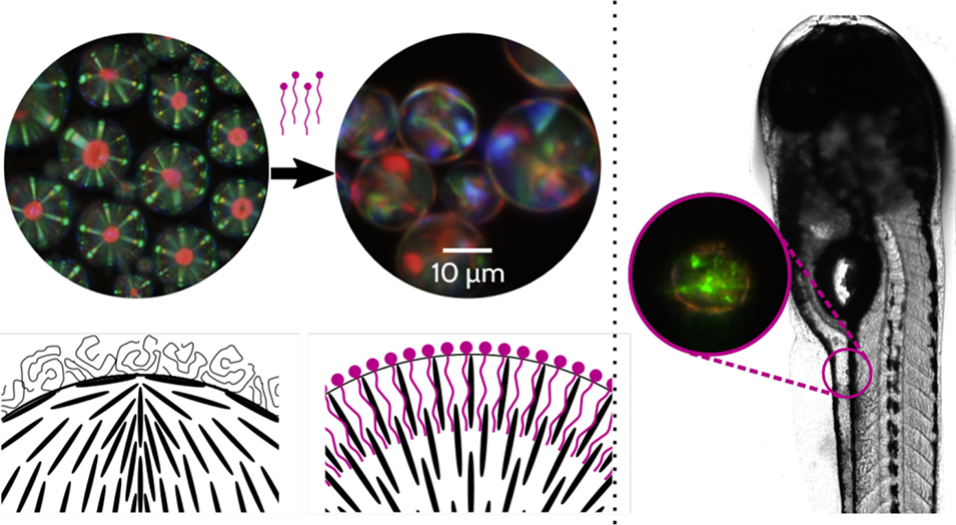

Biosensing using liquid crystals has a tremendous potential by coupling the high degree of sensitivity of their alignment to their surroundings with clear optical feedback. Many existing set-ups use birefringence of nematic liquid crystals, which severely limits straightforward and frugal implementation into a sensing platform due to the sophisticated optical set-ups required. In this work, we instead utilize chiral nematic liquid crystal microdroplets, which show strongly reflected structural color, as sensing platforms for surface active agents. We systematically quantify the optical response of closely related biological amphiphiles and find unique optical signatures for each species. We detect signatures across a wide range of concentrations (from micromolar to millimolar), with fast response times (from seconds to minutes). The striking optical response is a function of the adsorption of surfactants in a nonhomogeneous manner and the topology of the chiral nematic liquid crystal orientation at the interface requiring a scattering, multidomain structure. We show that the surface interactions, in particular, the surface packing density, to be a function of both headgroup and tail and thus unique to each surfactant species. We show lab-on-a-chip capability of our method by drying droplets in high-density two-dimensional arrays and simply hydrating the chip to detect dissolved analytes. Finally, we show proof-of-principle *in vivo* biosensing in the healthy as well as inflamed intestinal tracts of live zebrafish larvae, demonstrating CLC droplets show a clear optical response specifically when exposed to the gut environment rich in amphiphiles. Our unique approach shows clear potential in developing on-site detection platforms and detecting biological amphiphiles in living organisms.

## Introduction

Widely used in digital display applications,^1^ liquid crystals (LCs), ordered fluid phases formed from strongly anisotropic molecules, have become increasingly popular for use in chemical, physical, and biological sensing applications.^2−13^ Many of the same qualities that have lent themselves to their use in displays—extreme sensitivity to alignment-inducing conditions, rapid switching and response times, and a clear optical response—make them well-positioned for their use in sensing applications. Most sensing research to date has used nonchiral nematic LCs (NLCs),^3−6,8,14,15^ looking at either the switching between alignment configurations or the changes in the LC phase caused by the adsorption of an amphiphile^2,16,17^ or by the infiltration of a contaminant such as a volatile gas.^7,10,18−21^ Simple prototype sensors have been developed based on the use of the LC either as a primary or as a secondary sensing component.^2,22^ These visualize the presence or absence of an antigen through a switch of alignment that then becomes reflected in a change in birefringence. A limiting condition is that NLCs function normally as a binary switch, transitioning between a bright “on” state and a dark “off” state. Only in some extreme cases can transient states of tilted alignment be observed, making intermediate concentrations distinguishable, but a complete change of LC alignment is typically observed at concentrations well below the critical micelle concentration (CMC):^17,23^ once full switching has occurred, it is difficult to extract further information, if any, from an NLC interface.^22,24^

A less explored but equally interesting direction is the use of chiral nematic liquid crystals (CLCs), also known as cholesteric liquid crystals, for sensing applications. CLCs, in addition to the alignment-dependent birefringent properties of NLCs, have a helical modulation in their orientation. These are usually formed by mixing a chiral molecule (often called a “dopant”) into a nematic phase, with the final equilibrium pitch *p*_0_ being determined by the “helical twisting power” (HTP) of the chiral dopant and its concentration [*c*]: .^25^ While characteristic helical ordering and textures can become evident even with low concentrations of added dopant,^4,26,27^ high dopant concentrations (typically between 25 and 35% w/w) are usually necessary to give rise to chiral phases with strongly reflected colors in the visible range.^20,28−31^ These reflected colors, arising due to Bragg reflection, are a function of their helical arrangement and the viewing angle,^28,31^ analogous to the structural colors found in bird wings and in certain fruits.^32^ Another notable aspect of CLCs is that, because the obtained colors are not solely due to birefringence, one can observe these striking colors without the aid of polarizers. This leads to CLCs to being more readily incorporable into an eventual device with an output that can be easily interpreted by lay technicians.

One of the oldest uses of CLCs in sensing is in quick-response thermometers, where changes in color correspond to a change in temperature.^33^ Several other sensors have incorporated CLCs in order to detect the presence of volatile organic compounds,^20,21^ for pH,^34^ for the detection of antibody–antigen binding events,^35^ for humidity,^13,36^ and into rubbers for strain sensing.^11,12^ Much less studied, however, are the effects of adsorption of different types of biomolecules to an CLC interface, and in particular the use of CLCs for sensing of surface active agents, especially amphiphilic biomolecules such as fatty acids and lipids. These are molecules that are key biological components of the (intra)cellular membrane, metabolic pathways and that play a crucial role in various medically relevant conditions^2,24,37^ as well as possible contaminants in biodiesel production.^22^ CLC droplets are drawing increasing interest for biological and chemical sensing applications, the impetus arising from a study in 2016 by Lee et al. on the sensing of synthetic surfactants by using CLC droplets and the use of these droplets to sense pH.^30^ While ordinary nonchiral nematic liquid crystal droplets will show a transition from a tangential/planar anchoring configuration to a homeotropic/normal anchoring, evidenced by a change from a bipolar/multipolar structure required by the Poincaré–Hopf theorem to a Maltese cross,^23,38^ the transition in CLC droplets was shown to go from a Frank–Pryce texture with uniform helical ordering to a diffuse, “chicken skin”, multidomain texture.^30^ However, the use of CLCs for biological sensing is a field with much untapped potential for exploration: while much research has looked more into answering, “Is something there?”, less has looked into instead answering “Is *some thing* there?”, looking at whether an LC interface can actually tell us about what is present rather than merely indicating the presence of something.

In this paper, we show that micrometer-scale CLC droplets, selectively and sensitively, optically respond to amphiphiles in *in vitro* as well as *in vivo* settings, underlining their potential for rapid biological sensing. By exposing CLC droplets (*p*_0_ ∼ 650 nm) to different species (surfactants, fatty acids, and lipids) and concentrations (order of micromolar to millimolar) of amphiphiles, we observe clear optical differences with each species, providing a unique “optical signature”, including molecules with the exact same carbon tail but differing headgroups. In order to make our sensing platform robust, easy to use, and easily transportable, we stably deposit the CLC droplets on a glass surface at high densities in the dried state. These compact arrays of CLC droplets elicit similar optical responses when rehydrated with an amphiphile-containing sample, with the response time being as short as seconds for higher amphiphile concentrations. Lastly, we demonstrate proof-of-principle *in vivo* testing by gavaging live zebrafish larvae with the droplets, showing the potential of CLC droplets to detect amphiphiles within the intestine such as, for example, products of microbial fermentation^39,40^ or metabolites that may be indicative of microbial dysbiosis or inflammatory processes.

## Results and Discussion

### CLC Droplets Can Sense Diverse Amphiphiles Qualitatively and Quantitatively through Their Distinct Optical Responses

**Figure 1:**
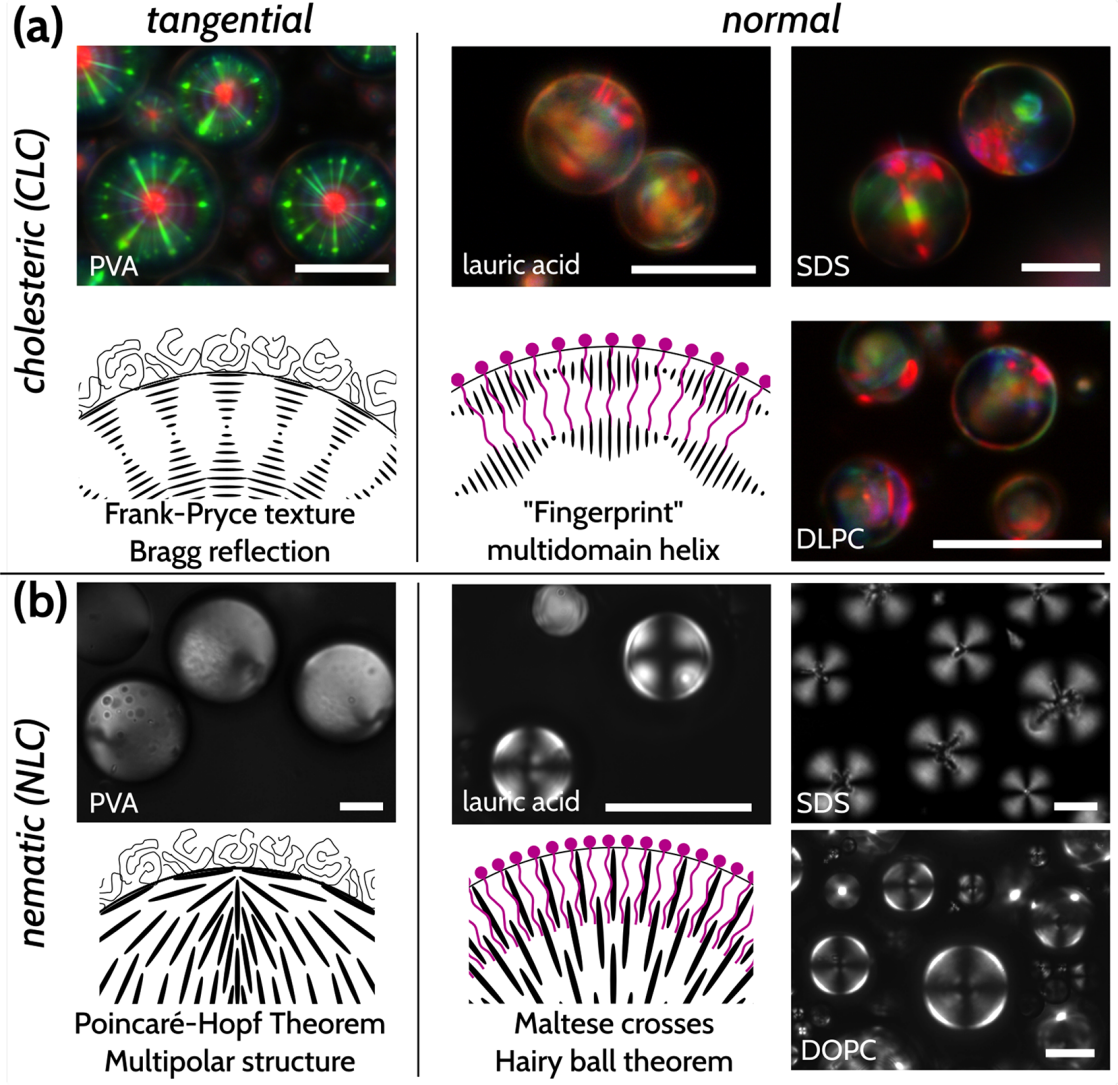
CLC droplets show differences in texture when exposed to different amphiphiles, while NLC droplets exposed to the same or similar materials look structurally similar. (a) Droplets of a red-reflecting CLC mixture prepared in buffer solutions containing different amphiphiles showed remarkably different apparent textures. While the droplets in PVA showed the expected Frank–Pryce texture, the fingerprint textures when using three different amphiphiles were markedly different. (b) In contrast, droplets of the common nematic LC 5CB showed an as-expected multipolar texture when prepared in 5CB, but the Maltese crosses obtained with each of the different amphiphiles did not show appreciable differences. Samples were prepared in 0.2% w/w PVA solution and in solutions of a synthetic anionic surfactant (6.0 mM SDS), a long-chain fatty acid (5.0 mM lauric acid), and a phospholipids (40 μM DOPC or 100 μM DLPC). Micrographs in panel a are in reflection mode with crossed linear polarizers; micrographs in b are in transmission mode with crossed linear polarizers. Schematics are a two-dimensional projection of an otherwise three-dimensional structure. Scale bars 25 μm.

Our first question was to determine whether or not CLC droplets could systematically sense the presence of different amphiphiles and if these responses could be differentiated between different amphiphilic molecules. For our experiments, we used a mixture of 35% w/w CB15 chiral dopant in the eutectic liquid crystal blend RO-TN 407, a red-reflecting CLC mixture (pitch ∼650 nm) that remains in the chiral nematic phase over a broad range of temperature, at least across 0–45 °C. We prepared a bulk aqueous dispersion of the LC phase using different amphiphile solutions of interest, the structures of which are shown in Figure S1. With this method, we were able to obtain a size distribution of droplets in the micrometer range, with an average diameter 33 ± 11 μm (mean ± standard deviation; Figure S2.) We used poly(vinyl alcohol) (PVA), a polymer that possesses surface-stabilizing qualities while leaving the LC orientation with respect to pure water undisturbed,^41^ as our negative control so that the LC will be oriented tangentially/planarly with respect to the water interface. We tested several different synthetic and naturally derived amphiphiles (sodium dodecyl sulfate (SDS), a strong anionic surfactant which is well-characterized in LC-based systems; lauric acid (LA), a long-chain fatty acid with identical alkyl tails to SDS; and different phospholipids as the target sensing materials. It is worth noting that PVA does possess some amphiphilic character, especially at lower grades of hydrolysis, particularly due to the combination of the hydrophilic pendants and hydrophobic tail.^42,43^ In this work, however, we use “amphiphile” as a shorthand to refer to amphiphilic molecules that switch the alignment of the liquid crystal, which PVA does not do.^41^Figure 1a shows examples of the final textures in the presence of each of these amphiphile solutions. The situation with planar/tangential anchoring is as expected, with the well-ordered Frank–Pryce texture producing strong interdroplet reflections characteristic of the ordering. Interestingly, the use of different, homologous amphiphiles produced different fingerprint textures between each of the different amphiphiles used. While some degree of variation can be expected due to the degeneracy of the alignment of the fingerprint texture, the apparent colors and patterns we observe are markedly different between each system.

As a point of comparison, we saw that with droplets of NLCs like 5CB (Figure 1b), while the negative control (using PVA) produced the typical planarly/tangentially aligned texture, with surface defects imposed by the geometry (per the Poincaré–Hopf Theorem, requiring a minimum of two defects at the poles^44^), NLC droplets that adopted a homeotropic/normal configuration upon exposure to SDS, fatty acids (lauric acid, LA), and phospholipids (such as 1,2-dioleyoyl-*sn*-glycero-3-phosphocholine, DOPC) showed the typical and expected “Maltese cross” with a single, prominent point defect at the center (governed by the hairy ball theorem).^16,45^ Using fluorescent lipids further confirmed that the tangential-to-normal switching was caused by a monolayer of lipid molecules adsorbing to, stabilizing, and inducing normal anchoring at the interface (Figure S3), which has been independently confirmed by other works.^31^ This switching process in nematic droplets is partly due to the complex energy landscape of the LC alignment. For many common nematic liquid crystals (such as 5CB), the energy deformation associated with the Poincaré–Hopf multipolar tangential texture is a bend deformation, which has a higher energetic cost than the splay deformation present with the “hedgehog” we see with normal alignment,^46^ but the interfacial tension associated with normal alignment in the absence of amphiphiles is higher than that of tangential alignment,^23^ which can help to explain the droplets in the absence of amphiphiles adopting a tangential configuration. Ultimately, however, we did not see appreciable differences between the final Maltese cross textures that developed, regardless of whether SDS, fatty acids, or phospholipids were used for switching.

**Figure 2:**
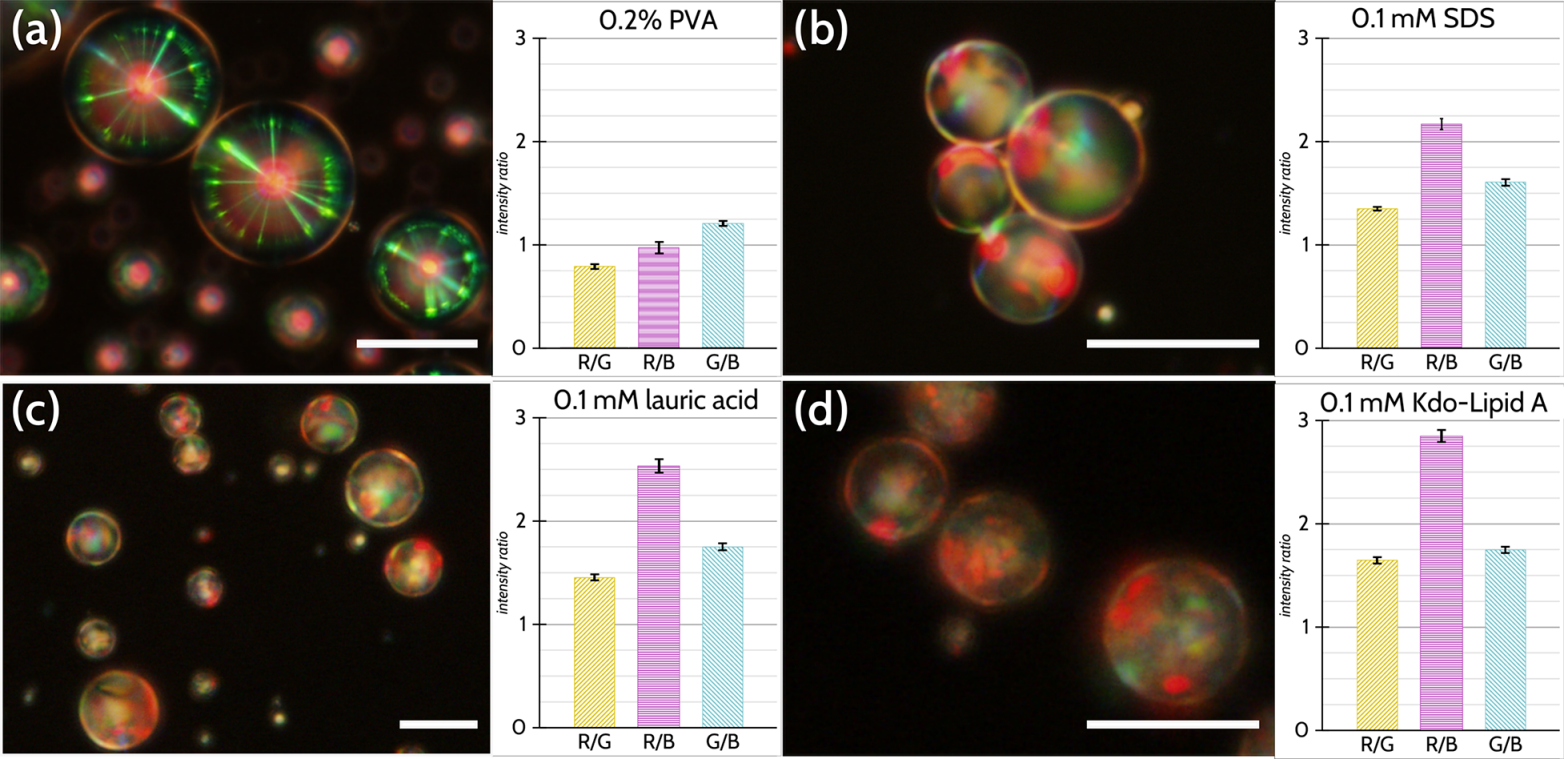
CLC droplets can show quantifiably different responses to the same or similar concentrations of different amphiphiles. (a) Droplets of red-reflecting CLC suspended in an aqueous solution of 0.2% w/w poly(vinyl alcohol) (PVA) in Tris (pH 7.4) buffer. The Frank–Pryce texture produced by tangential/planar anchoring of the LC at the surface is clear and dominant, with the single red-reflecting central spot accompanied by strong interdroplet reflection (the green “starburst” pattern; larger densities of droplets will lead to more interdroplet reflections and increase the green reflection). (b–d) Switching induced with equal molar concentrations (0.1 mM) of (b) SDS, (c) lauric acid, and (d) Kdo2-Lipid A. Scale bars 25 μm. Error bars represent the standard error of the mean for each sample (*n* > 50 droplets).

Armed with the preliminary observations in Figure 1, we sought to investigate if these differences were quantifiable and if we could systematically establish differences between different amphiphile solutions. To do so, we prepared dispersions of CLC droplets in different solutions of amphiphiles and captured POM micrographs of the resultant droplets. We quantified the optical response by obtaining the average intensities of the three primary color channels (red, green, and blue) of individual droplets. In order to eliminate the influence of photography parameters, such as background illumination, exposure time, and light intensity, rather than using the color intensities themselves, we instead analyzed the intensity ratios (R/G, R/B, and G/B) to study the differences between the responses generated from different amphiphiles (see Methods for details). This straightforward analysis gave us a unique “optical signature” for each amphiphile, as shown in Figure 2.

For each of the four environments, at least one color ratio showed a statistically significant difference with each of the other three amphiphiles (Figure S4). We notice that, in the case of PVA (Figure 2a), the dominant color in the image is green, which is reflected in the comparatively high G/B ratio and the very low R/G ratio. The high degree of green reflection in Figure 2a is a consequence of interdroplet reflections^28^ from the Frank–Pryce structure: with a lower droplet density, the green reflections become suppressed. While PVA is amphiphilic, it is not a surfactant: it merely serves to stabilize the droplets without changing their alignment compared to the alignment in pure water or buffer solution because of the high interfacial tension between an LC and water.^23,47,48^ As for the other three amphiphiles, the colors and textures we observe look visually different, with more colors visible overall and a more “muted” appearance, the Frank–Pryce structure having transitioned into a “fingerprint” with different scattering domains. While they show similar trends (with the R/B ratio being the highest in each), there are statistically significant differences (*p* < 0.05) in the R/G and R/B ratios for these three samples. This thus shows that color ratio analysis can be used as a distinguishing tool to differentiate between amphiphiles.

**Figure 3:**
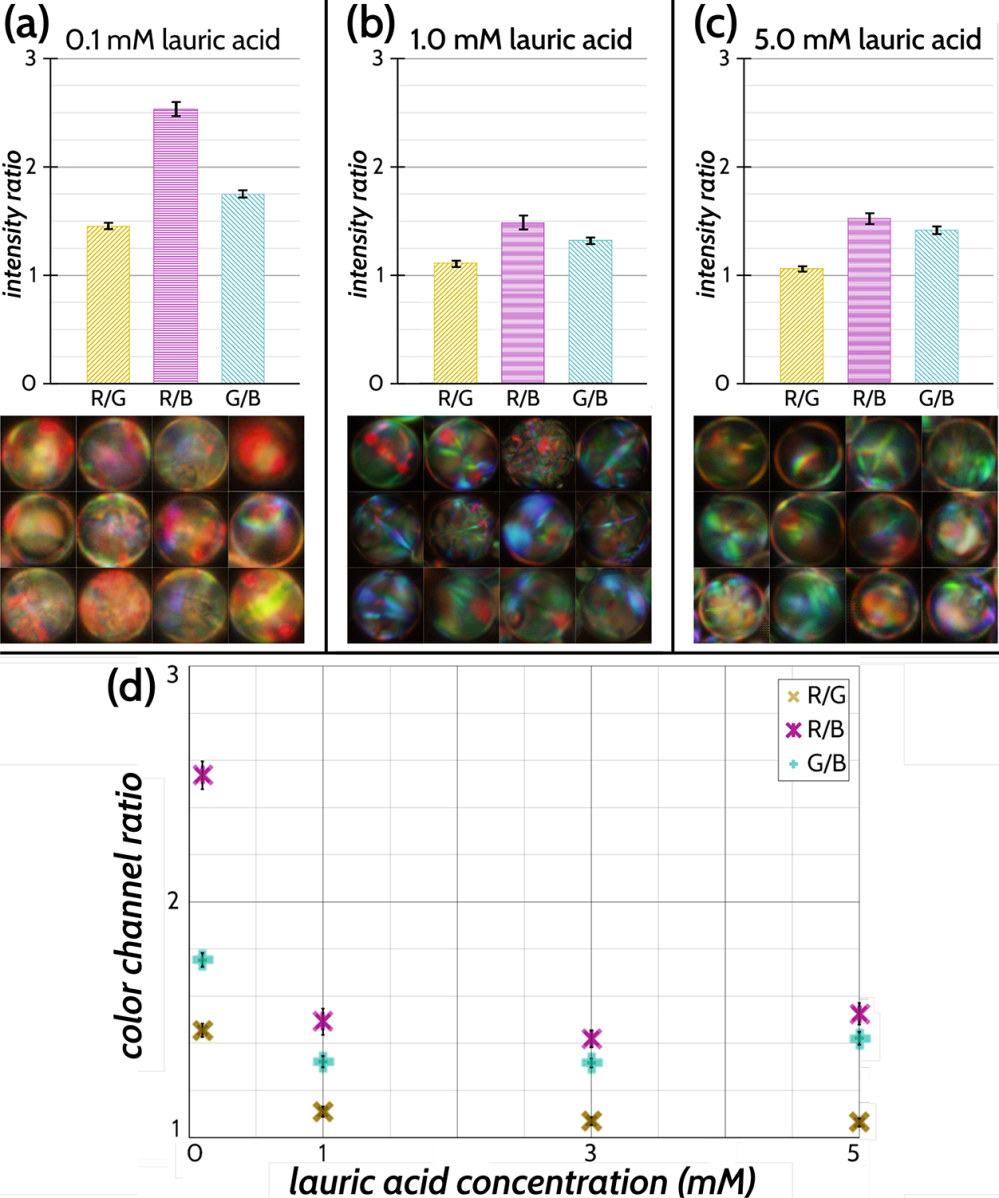
CLC droplets can show differing optical responses to different concentrations of the same surfactant. We prepared droplets in solutions containing (a) 0.1, (b) 1.0, and (c) 5.0 mM lauric acid and saw quite clear differences in the colors of the droplets, with samples prepared at lower concentrations of lauric acid visually appearing redder and higher concentrations appearing greener. By analyzing large numbers of individual droplets (samples of which are shown below the respective histograms), we were able to use the color channel ratios to distinguish between the concentrations, with the most pronounced differences being between 0.1 mM lauric acid and the other two samples. (d) Plot of the color ratios as a function of concentration of lauric acid, clearly showing that concentration does not have a strong effect on the color ratios on approaching the saturation point of the interface. Micrographs viewed between crossed linear polarizers. Error bars represent the standard error of the mean (*n* > 85 droplets).

While we can use color channel ratios to distinguish between materials, what about using them to distinguish between different *concentrations* of the same amphiphile? We examined these effects between different concentrations of lauric acid, the results of which are shown in Figure 3. Visually, we clearly notice that the sample prepared in 0.1 mM lauric acid (Figure 3a), appears redder than those prepared in both 1.0 mM and 5.0 mM lauric acid solutions (Figure 3b, c). As shown in Figure 3d, this is reflected in the R/G and R/B ratios being higher for this solution than the other two, which appear more green and blue (as is reflected in the lower ratios and the more moderate G/B ratios). There is enough of a statistically significant difference for the color ratio values to distinguish between lower (0.1 mM) and higher (1–5 mM) concentrations of lauric acid, but not between the higher concentrations. As will be clear later, these values correspond to the concentrations below and above the critical micelle concentration (CMC) of the amphiphile. An expanded series of concentration-dependent effects for all the amphiphiles used in the study is presented in an analysis matrix in Figure S5, which shows we can distinguish between different amphiphiles as well as different concentrations of the same amphiphile, i.e., we find differences between most samples for at least one color ratio. In general, the differences we see between lower concentrations of amphiphiles (e.g., between 0.1 and 0.6 mM SDS and between 0.1 and 1.0 mM lauric acid) are more appreciable than those between higher concentrations. As will be discussed shortly, we believe this to be a consequence of the surface coverage and the relationship to the CMC of each of the amphiphiles, where the main determining factor is how saturated the surface is with the amphiphiles.

We additionally checked the impact of droplet size on the color ratio values. While the absolute intensities of the color channels did increase with the size of our droplets, we did not see significant differences in the color ratios as a function of droplet size. By using lauric acid as the sensing amphiphile, we saw that the final color channel ratios were largely independent of the size of the droplets (Figure S6). While birefringent color is a function of path length and, thus, size,^49^ the dominance of the structural color reflection significantly reduces the effects of birefringent colors in case of CLC droplets.

How are these structurally similar amphiphiles and different concentrations of them able to give distinct optical responses? It was noted by Popov et al. that lipids of different handedness as well as nonchiral amphiphiles can change the equilibrium pitch of CLC films.^4^ This was additionally demonstrated for nonchiral materials, such as the surfactants and fatty acids we use in this work, where the final equilibrium pitch of the fingerprint texture differs from the measured pitch using a chiral dopant alone: in their work, by using a long-pitch CLC, the fingerprint spacing when using air as the homeotropic anchoring substrate differed from when a nonchiral surfactant (such as SDS and tetraethylene glycol monooctyl ether, a nonionic surfactant) was used to induce homeotropic anchoring.^27^ While their work was typically performed with amphiphile concentrations well above the CMC, it gives us a clue that one of the main determining factors is the interaction of the tails with the LC changing the twisting of the CLC due to the insertion of the hydrocarbon tails into the LC material:^50,51^ since our LC materials are effectively oils, the hydrocarbon tails will preferentially insert themselves into the oil phase to minimize energy. We would expect that identical tails should produce similar distortions in the LC interface, part of the reason we chose to study lauric acid and SDS, but we found this not to be the case. We thus reasoned that the behavior of different surfactant molecules at the LC–buffer interface must be significantly different, which would likely get reflected in their interfacial tension and the associated surface coverage parameters.

To get an idea of how the surface coverage differs between lauric acid and SDS as our model amphiphiles, we performed pendant drop measurements to obtain the surface tension (against air) at various surfactant concentrations. We can then use the behavior of our amphiphiles at such a hydrophilic–hydrophobic interface to get an idea of how they pack and arrange at the LC–water interface over the short durations of our experiments; additionally, we can determine the critical micelle concentrations of each of the amphiphiles in the buffer solutions using this method.

**Figure 4:**
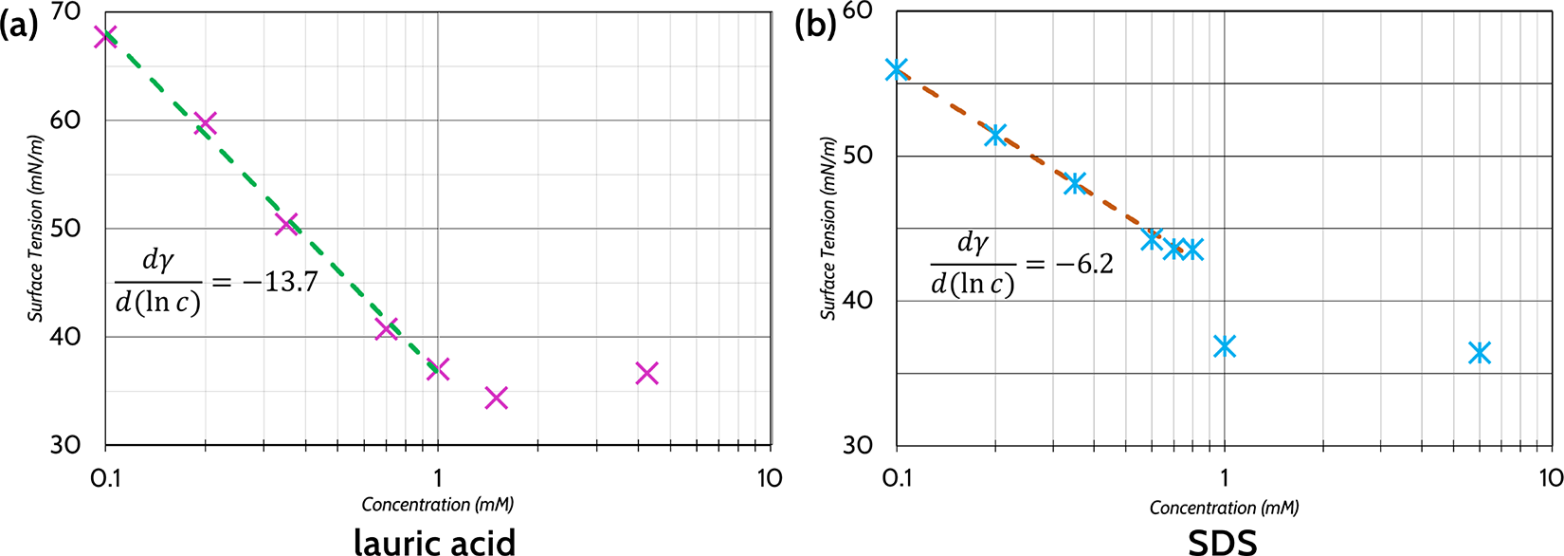
Lauric acid and SDS show different surface behaviors up to their critical micelle concentrations, demonstrating that they pack differently at the surface. Surface tension measurements performed on (a) lauric acid and (b) SDS solutions prepared in Tris buffer (pH 7.4) using a pendant drop technique. We found lauric acid to have a critical micelle concentration (CMC) of ∼1.3–1.5 mM in the buffer solution, while SDS had a CMC of ∼0.9 mM. Fits to  were performed on the interval clearly below the CMC, obtaining  with *R*^2^ = 0.996 for lauric acid and  with *R*^2^ = 0.995 for SDS. This suggests that SDS molecules have a generally higher surface area per molecule coverage.

Panels a and b in Figure 4 show plots of the surface tensions of both lauric acid and SDS solutions across the relevant concentration range. We note that while the reported critical micelle concentration of SDS is 8.2 mM in pure water,^23,52^ it is markedly lower (∼0.9 mM) in our case: this is likely due to the specific buffer conditions and presence of salts which screen the charges of the headgroups.^53^ More importantly, we obtained a strong linear fit to ln*c* (*R*^2^ = 0.996 for lauric acid and 0.995 for SDS) for the values below the CMC in case of both the amphiphiles (1.0 mM and below for lauric acid; 0.8 mM and below for SDS). The obtained value of the slope () for lauric acid (13.7) is well over double that obtained for SDS (6.2). We then used the Gibbs adsorption law to relate this surface coverage parameter Γ to the interfacial tension as a function of concentration γ(*c*), , allowing us to quantify how well the surfactant molecules cover the interface. Inserting the obtained slope values in the equation, we obtain Γ_LA_ = 1.69 × 10^21^ m^–2^ and Γ_SDS_ = 7.67 × 10^20^ m^–2^. The surface coverage of the two molecules is thus markedly different, with Γ_LA_ over double that of Γ_SDS_. This is consistent with the logic that SDS has a larger and a more strongly and permanently charged headgroup which promotes a stronger electrostatic repulsion between neighboring molecules, resulting in an overall lower surface density. While these calculated quantities are likely not the exact packing numbers at the LC–water interface, we can expect similar effects especially over the short time scales of the sensing experiments. The differences in the surface packing density likely lead to differences in how the LC director becomes distorted, both in terms of the final pitch of the CLC and the formation of domains and disclinations in the aligned structure, so surface coverage and assembly of the surfactant at the LC–water interface could be the primary cause of the differences in the colors we see. The different packing density of amphiphiles at the interface is dictated by the steric and electrostatic repulsion from the headgroups, affecting the placement and orientation of molecules at the interface. The phenomena we observe are likely not a direct consequence of the identities of the specific chemical groups present in the molecule, but an effect of both the physical size differences of the headgroups (the carboxyl group of lauric acid is less than half the mass of the sulfate headgroup of SDS) plus the effects of the ionic charges increasing the effective excluded volume around each headgroup (SDS readily dissociates in solution; lauric acid does not do so as readily). These parameters affect the packing density, with the combination of both the strong ionic charges on the headgroup and the larger headgroup mass contributing to a “looser” packing of SDS molecules at the interface, while lauric acid can pack more tightly (and have more molecules covering the surface) because of its smaller headgroup and weaker electrostatic repulsion. It could be conceivable that a molecule with exactly the same headgroup mass and charge may produce a similar signal, though there may be additional effects resulting from the headgroup affecting the conformation of the aliphatic tails. There may also be differences in the resultant conformation of the aliphatic tails upon insertion into the LC layer, but we find that such an analysis could be difficult to perform experimentally and is possibly beyond the scope of this paper. We conclude that, even if the surfactant tails may have similar effects on the distortion of the CLC orientation, the effects of the headgroup mass and charge are more significant determining factors in the final optical signature. The fact that surface coverage is a dominant factor in the final orientation of the LC is additionally suggested by the less significant differences in color ratio intensity when samples are close to or above the critical micelle concentration (at which surface coverage is more or less saturated), as seen in Figure 3d.

### CLC Droplets Can Be Dried and Rehydrated to Quantitatively Sense Amphiphiles and to Visualize the Switching Process

**Figure 5:**
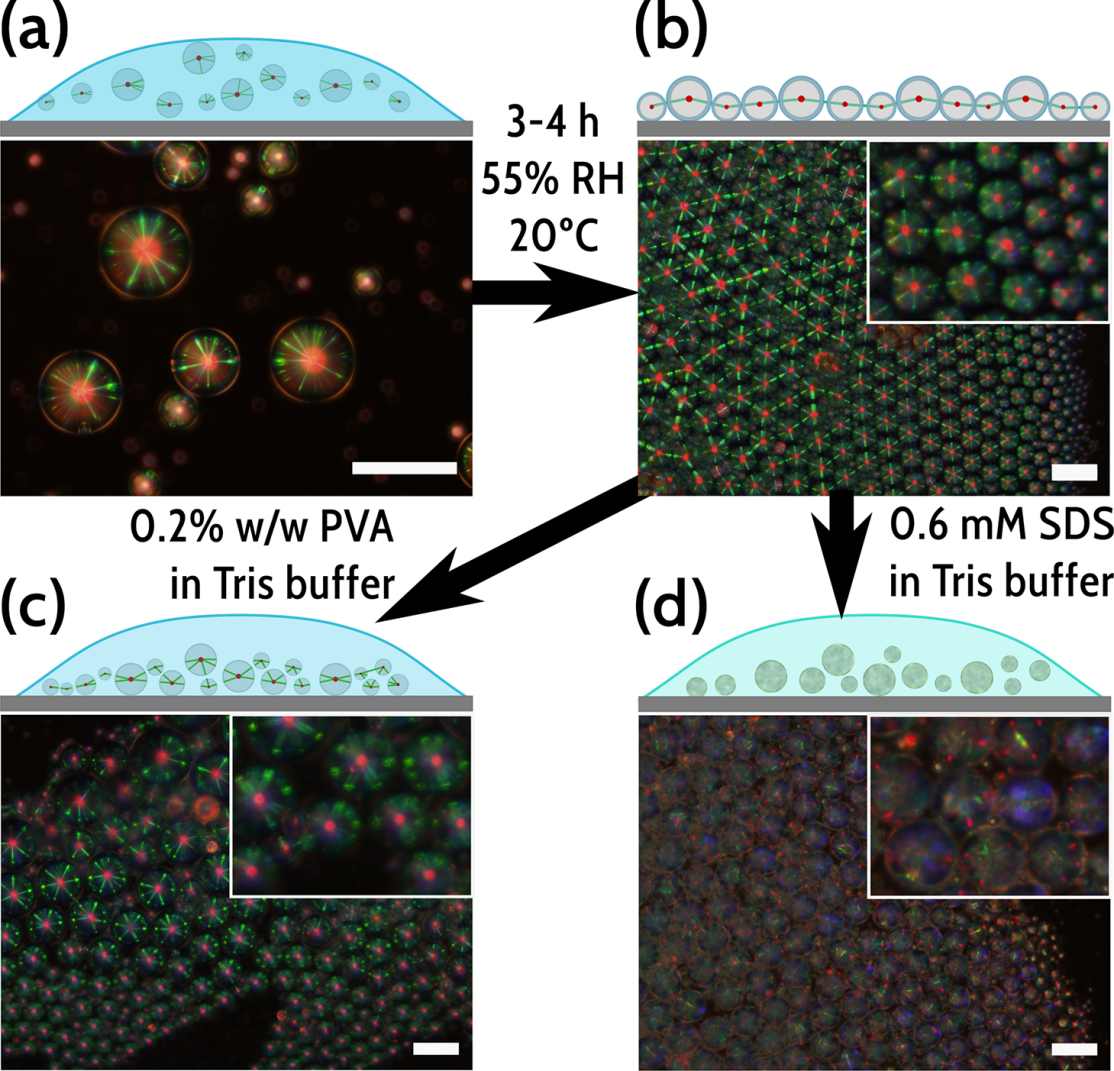
Droplets dried on glass slides in the presence of PVA will still remain responsive to the later addition of amphiphiles. (a) Dispersion of CLC droplets prepared in PVA buffer solution, showing the expected texture. (b) After depositing 10 μL droplets of solution on the glass slide and drying under ambient conditions for at least 3 h, we create a two-dimensional quasicrystalline array of droplets, the characteristic Frank–Pryce structure clearly preserved after the drying process. (c) Carefully rehydrating such an array anew with fresh PVA solution does not change the alignment of the droplets, while still preserving the Frank–Pryce structure. (d) Rehydrating the array with a solution of SDS, for example, induces a rapid change of alignment of the droplets from the Frank–Pryce structure to the fingerprint textures observed in Figure 2b–d. Micrographs were viewed in reflection mode between crossed linear polarizers. Scale bars 50 μm.

During our control experiments, we noted that when depositing droplets suspended in solutions of PVA on glass slides and allowing them to dry under ambient conditions, a large number of droplets could survive the drying process, forming compact, quasi-crystalline, two-dimensional arrays of planarly aligned droplets with very obvious Frank–Pryce textures visible and strong interdroplet reflections. We were able to consistently dry droplet suspensions prepared from 0.2% w/w PVA solutions without significant droplet loss. We thus investigated whether we could rehydrate these arrays of droplets with amphiphile solutions and observe visible switching with the amphiphiles of interest, a step that would make our droplets more amenable for lab-on-a-chip capability. Figure 5 shows the drying and rehydrating process for such an array.

We found that the drying and rehydration was quite robust, with dried arrays of LC droplets able to survive on the order of weeks while still maintaining their responsiveness. However, the sensitivity of these arrays to amphiphiles was reduced compared to when they were in bulk: while the Frank–Pryce texture of CLC droplets would readily be lost even at nanomolar concentrations of the amphiphile (50 nM for SDS; 10 nM for LA and DLPC) in bulk solutions, significantly higher concentrations (typically at least 100 μM) were needed to switch the droplets dried on glass slides (Figure S7a, b). Once dried, the droplets were not responsive within experimental time scales to amphiphile concentrations that would switch them in bulk samples (Figure S7c, e). Thus, in the current state, the concentrations of amphiphiles necessary to induce an optical response in our dried sensor arrays are quite high, on the order of mM. The likely cause of this reduced sensitivity is, indeed, the PVA present. To dry the droplets in a manner that prevents their collapse onto the glass slide or coalesence with each other, we needed to add PVA to the buffer: drying droplets without PVA would result in their collapse onto the glass slide. At the same time, the protective coating formed by the PVA polymers around the LC droplets can impede the adsorption of amphiphiles at the interface. We used a relatively high PVA concentration of 0.2% w/w, corresponding to ∼0.1 mM, in order to stabilize the droplets during the drying process. Additionally, PVA molecules are considerably larger compared to the size of the amphiphiles detected (∼60× larger), further shielding the CLC droplets from amphiphile exposure. Lower concentrations of PVA were tested, with some success attained with 0.02% w/w PVA, but drying at lower concentrations often did not keep the droplets fully intact during the drying process for eventual sensing. The adsorption of polymers at interfaces and the displacement of polymers at an interface by monomers is a known phenomenon, where a nonionic polymer can be displaced from an interface by the adsorption of small surfactant molecules.^54,55^ Depending on the size of the surfactant and of the polymer used, the polymer adsorbed at the given interface can be dislodged by adsorption of the surfactant,^54^ which then becomes optically reflected in the final texture.

**Figure 6:**
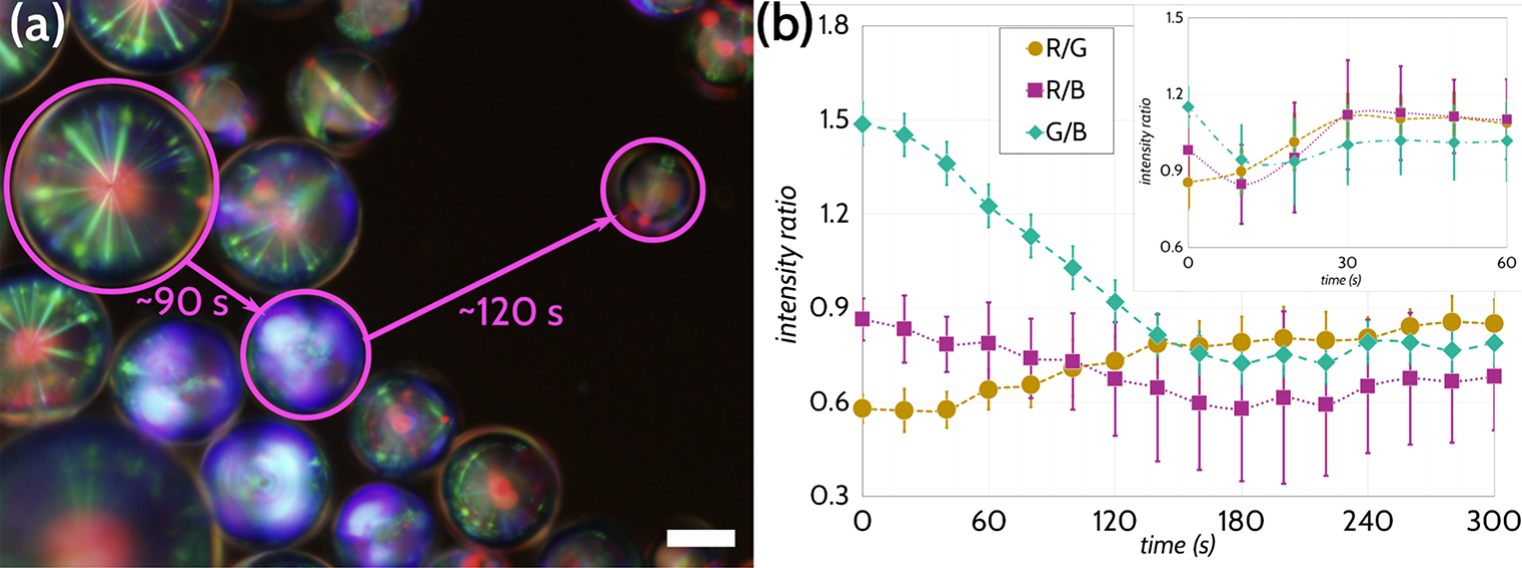
Droplets during the switching process can exhibit complex dynamics in their optical response. (a) Sample of CLC droplets exposed to 1.0 mM SDS in Tris buffer showing the different stages of the switching process, ranging from fully tangentially/planarly aligned, where a single red reflection and a starburst pattern from interdroplet Bragg reflection is evident; a transient metastable texture, where the helix axis is tilted off-vertical en route to normal/homeotropic alignment, the result being a blue-shifted Bragg reflection; and fully normally/homeotropically aligned, where we instead see a multidomain reflection without a single, prominent central reflection. Smaller droplets and droplets not shielded by others tended to switch more quickly, though the response time from the onset of switching to the final fingerprint texture was generally the same. Viewed between crossed linear polarizers. Scale bars 25 μm. (b) Over the time of the switching process with both 0.6 mM SDS and 6.0 mM SDS (inset), the color ratios likewise change, with the G/B ratio declining sharply and the R/G ratio increasing with time. These effects are likely a consequence of the loss of the strong interdroplet reflections with the Frank–Pryce textures, which typically appeared green. A peak in the blue intensity (reflected in the dip of the G/B and R/B ratios) is due to the “blue fog” that appears during the switching, though this trough then dissipates with time. The sample exposed to 6.0 mM SDS showed a similar behavior, though over a much shorter time scale, reaching an equilibrium state more quickly. The graph was generated by randomly selecting 10 droplets from the solution and analyzing the color channel intensities every 20 s (for 0.6 mM SDS) or every 10 s (for 6.0 mM SDS). Error bars indicate standard deviations.

The use of on-chip switching also allows us to visualize the switching of the droplets as it occurs, as presented in Videos S1–S4 as well as in Figure 6a. We can see that the droplets initially exhibit a uniform Frank–Pryce structure before transitioning to an intermediate “blue fog”, likely a function of a transient shift of the structural color reflection in the droplet as the LC orientation transitions from tangential to normal,^56^ before finally adopting a metastable normally aligned “fingerprint” texture (which itself is partly due to the metastable textures possible when inducing the LC to change anchoring conditions^24^). We analyzed this dynamic process, as shown in Figure 6b, as a function of time, where we plotted the change of color intensity as a function of time and see clear trends corroborating with the visual observations. Over time, the green reflection notably becomes suppressed, likely corresponding to the loss of interdroplet reflections, and is reflected in the sharp decrease of the G/B ratio. Additionally, at concentrations of amphiphiles where the differences between color signals are not particularly distinct between each other (such as Figure 3b, c, which both sit above the determined CMC of lauric acid in the buffer solution), we can use the time it takes to switch the droplet alignment as an alternate way to distinguish between concentrations of amphiphiles, as indicated in Figure S8, where we can distinguish between above-CMC concentrations of SDS by using the time it takes for droplets to fully switch to the fingerprint texture. This gives us an additional axis with which we can determine the quantity of a material present in the system and can thus be further explored in on-chip diagnostics and assays. Notably, the switching process using this system was one-way: once the droplets in the presence of PVA were switched by amphiphiles, the surfactants were irreversibly adsorbed to the interface and could not be displaced anew by the readdition of PVA buffer solution.

### CLC Droplets Can Be Used for *In Vivo* Sensing Of Amphiphiles From the Gut Microbiota of Zebrafish

With the above encouraging results, we further probed whether these droplets could be actually used for *in vivo* biosensing applications. The zebrafish (*Danio rerio*) is often used as a model vertebrate organism for the study of many processes because of its well-sequenced genome, fast development, and well-understood developmental behaviors. Importantly, amphiphilic molecules, especially short- and medium-chain fatty acids, are fermentation products from the gut microbiota. For example, butyrate is an important energy source for the intestinal cells (enterocytes) and has been shown to be associated with inflammation and dysbiosis (disturbances in the microbial composition).^39^ Here, we investigated whether CLCs could be used to visualize amphiphiles in the gut of a live organism. To this end, 5-day-old larvae (5 days post fertilization; dpf) were gavaged with CLCs and imaged both in transmission and reflection mode, as their entire gastrointestinal tract is open and functional at this point. Additionally, the zebrafish larvae being optically transparent at this age facilitates the localization and visualization of the LC droplets both in transmission and reflection mode.

**Figure 7:**
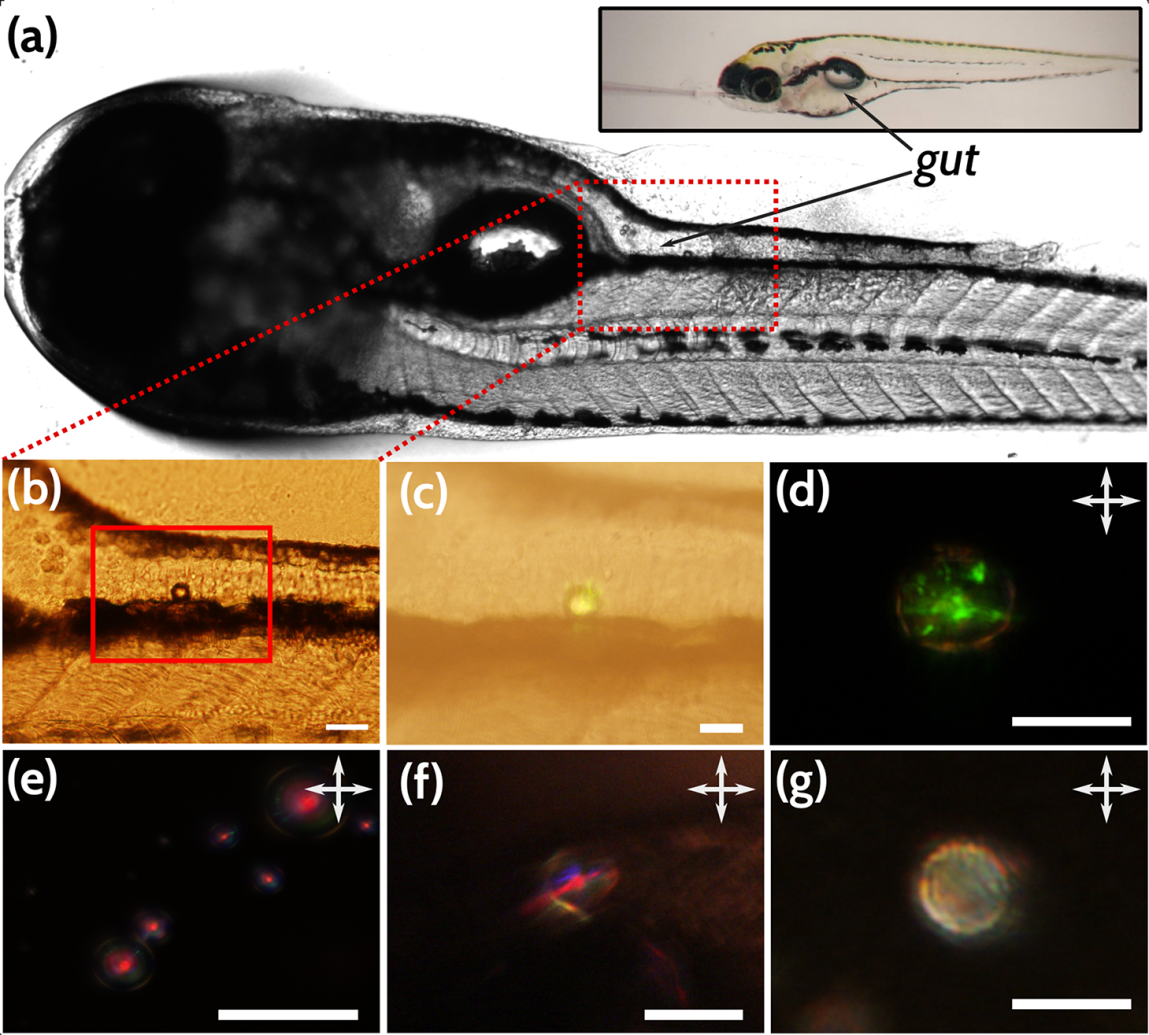
CLC droplets can detect amphiphiles within the zebrafish gut. A zebrafish at 5 days post-fertilization, gavaged with a dispersion of the CLC mixture suspended in a 0.2% w/w PVA in Tris buffer solution. (a) Zebrafish larva, with its gut clearly visible, viewed with a stereomicroscope. Inset: a demonstration of the gavaging process, with the glass capillary inserted into the oral cavity. (b) Inset showing a CLC droplet that has been lodged in the gut. (c) By using reflection mode microscopy without polarizers, we can see the droplet reflecting a green color. (d) Close-up in reflection mode microscopy between crossed polarizers clearly shows that the droplet no longer has the characteristic starburst texture associated with a planarly aligned CLC droplet and has a diffuse, scattering texture. This indicates the presence of an amphiphile, with the polarizers cutting out reflection from nonbirefringent surfaces. (e) *In vitro* control showing the characteristic Frank–Pryce texture when no amphiphiles are adsorbed to the LC interface. (f) Same texture seen in a droplet residing in the oral cavity of the fish instead of in the gut, indicating that the droplets are responding to amphiphiles within the gut rather than anything within the buffer solution. (g) Gut inflammation induced by growing the zebrafish larvae in soy saponin appears to produce a different response in the LC droplets, still with the absence of the central red reflecting spot. Scale bars in b, e, and f, 50 μm; c, d, and g, 25 μm. Panels d–g are viewed between crossed linear polarizers.

While the LC materials we use are themselves cytotoxic,^57^ and in light of our finding that droplets coated with PVA still remain sensitive to amphiphiles, we deduced that coating our LC droplet with PVA would make them more biocompatible while still being sensitive to the amphiphiles within the gut. In Figure 7, we illustrate how CLC droplets can be used to sense the presence of biologically relevant amphiphiles in the gut of a zebrafish. We embedded the fish in agar gel and gavaged them with 4 nL of the CLC droplet suspension (Figure 7a). Remarkably, the droplets lodged in the gut (Figure 7b–d) showed a complete lack of the central red reflective spot (indicative of the default tangential alignment/Frank–Pryce texture), but instead exhibited a diffuse reflection, associated with a switch because of surface active agents such as amphiphiles (Figure 2). The signal we observe is similar to the “chicken skin” pattern and texture described by Lee et al.^30^ that was also observed when amphiphiles were adsorbed to the interface of CLC droplets. While we observed considerable variations in the reflected colors for the few droplets that were successfully residing in the gut, each of them lacked a central red spot and showed a diffuse reflection, indicating the adsorption of surface active agents. Furthermore, due to the transparency of the zebrafish larvae (because of the lack of melanocyte development at this stage), the reflected signal was observable even without the use of polarizers (Figure 7c). Unsurprisingly, the signal becomes cleaner and more distinctive with polarizers (Figure 7d), as the polarizers cut out any reflection from nonbirefringent materials. Thus, we can conclude that amphiphilic molecules present in the gut, likely metabolic byproducts of the gut microbiota,^58,59^ are responsible for switching the droplet alignment. The aqueous medium in which the fish were grown did not switch the droplet alignment. A much better control was observed in the form of the LC droplets lodged within the oral cavity, clearly showing a single central red reflection (Figure 7f), which is characteristic of the reflection from droplets without adsorbed amphiphiles (Figure 7e). This further confirms that the droplet switch observed within the gut is indeed sensing the locally present amphiphilic compounds being produced within, most likely medium- to long-chained fatty acids rather than lipids, as the concentrations of lipids necessary to induce a response in PVA-coated droplets *in vitro* was found to be typically high. We also observe that the cartilage and bones of the zebrafish are, in fact, birefringent, much like we would expect from any aligned long-chained polymer or crystalline structure,^29^ but this birefringence is of much lower order than the LC^49,60,61^ (Figure S9). Many of the melanocytes also appeared birefringent, but their reflected textures were readily distinguishable from the signals from the CLC droplets (Figure S9).

Finally, we wondered whether we could see a difference between gut responses in different situations (healthy versus inflamed) by recording the optical response of LCs. For that, we grew the zebrafish larvae in a solution containing 0.5 mg/mL soy saponin, an inflammatory agent.^62^ Upon gavaging these treated fish with CLCs, we again saw a lack of the central red reflection that devolved into a “chicken skin” texture indicative of a multidomain orientation of the LC (Figure 7g and Figure S10). We additionally exposed some zebrafish larvae to butyrate, a short-chained fatty acid metabolite that is the product of anaerobic fermentation processes in the gut and a possible indicator of health,^39,63^ to see if the LC droplets would respond in such an environment. Treatment with butyrate showed differences in the CLC droplets compared to specimens in buffer solution, but also distinct from the those in the saponin-exposed zebrafish. While these droplets appear to show a different texture compared to the droplets visualized in healthy fish, the considerable variation within each group and the low sample size make it hard to draw a strong conclusion (Figure S10). The observed variation is not surprising given the natural biological variation between individual fish selected from different batches, the gavaging protocol not yet being fully optimized, and the significant variation in imaging conditions depending on the embedding of the zebrafish in the agar gel. Nonetheless, these preliminary results clearly show that we can harness the properties of CLC droplets for *in vivo* sensing applications and a full systematic characterization of these responses will be a centerpiece of future works.

## Conclusions

We have shown that cholesteric liquid crystals can be efficiently used to detect biorelevant amphiphiles such as fatty acids and phospholipids, down to nanomolar concentrations. We systematically quantified the optical response of different amphiphiles and showed that a simple parameter, i.e., ratio of the primary color channels, is sufficient to assign a unique signature to different amphiphiles. We showed that surface interactions, particularly the surface packing density, get dictated by the size and the chemical nature of the headgroups. The resulting differences in the packing density distort the LC director differently leading to surfactant-specific optical signatures. With the detection of amphiphilic analytes in portable on-chip settings as well as in highly complex biological environments such as the intestinal tract of zebrafish larvae, our easy-to-implement CLC sensors show high potential in biosensing.

Overall, our droplet-based sensors show statistically significant distinct responses over a wide range of amphiphiles, as presented in Figure 3 for lauric acid, Figure S5 for SDS, and in the overview matrix in Figure S4. In the absence of PVA coating, these CLC droplets are able to detect the analytes at nM concentrations (50 nM for SDS; 10 nM for LA and DLPC; see Figure S7). However, in the PVA-coated state, the limit of detection increases to ∼100 μM (both in the case of SDS and LA) in the current configuration. Additionally, the linear range (an interval over which the output signal behaves linearly with respect to the input) is often a useful means to measure the performance of a sensor, but generally only when the signal measured is an absolute intensity, such as fluorescence. In our case, since we use a color ratio rather than absolute intensity, it is not clear if a linear range is definable. What is clear, however, is that above the CMC of the analyte, it becomes more difficult to distinguish the responses between concentrations.

We see several ways of further improving our sensors. A priority would be improving the detection limit of the dried droplet arrays. The high affinity for polymers to adsorb at interfaces means that there may not be an ideal polymer that gives us both a high sensitivity of amphiphile detection while sufficiently stabilizing them for the drying process, though this remains a topic for future investigation. The reduction of sensitivity when using polymer stabilizers, on the other hand, could also be used as a useful feature to set the sensitivity of the LC to a specific level so that it can function as a threshold sensor, detecting the target analytes at only above a specific concentration. Additionally, we can look to optimize the drying to reduce the amount of necessary PVA, mostly by devising a more gentle drying process (such as better control of the humidity, minimizing air currents, and controlling the temperature) to ensure the survival of the greatest number of droplets. Alternately, we can investigate the use of wet assays and flow cells, similar to those employed by Bao et al. for on-chip peptide detection.^64,65^

We find that different amphiphiles present in a system will show qualitatively and quantitatively distinct behaviors with our LC droplets, though it is not yet clear, when given an unknown sample, if color ratios alone will be sufficient to distinguish between possible amphiphiles. Many commonly employed tests in biomedical sensing, however, are specific for single materials or narrowly defined classes of materials (such as rapid-antigen tests or hormone tests), for which CLC-based sensors may prove additionally useful to allow both for a quantitative axis of sensing and to provide rapid, clear output.

Despite the cytotoxicity of many LC mixtures,^57^ the CLC droplets did not have an adverse effect on the health of the zebrafish within the time duration of the conducted experiments of a few hours. This is very likely due to the PVA-coated surface of the CLC droplets and is a crucial advantage for *in vivo* sensing. Nevertheless, for future work, it is conceivable that biological analogues that form LC phases, such as cellulose nanocrystal dispersions,^66^ or more biologically friendly CLC-generating materials, such as cholesteryl ester mixtures,^67^ could be used as more biocompatible sensing materials. The capability to sense amphiphiles *in vivo* within the zebrafish additionally shows great promise in being able to rapidly detect and sense the presence of biologically relevant markers, such as amphiphilic short-chained fatty acids, which typically require complex set-ups or sophisticated equipment to detect.^59^ Other applications for *in vivo* sensing in fish include detecting how well feed is uptaken and metabolized, enabling for further optimization of animal husbandry.^58^ We envision incorporating CLC droplets into feed, either for imaging within the animal or for postanalysis after passing through the digestive tract: such an ingestion-and-retrieval strategy of microsensors to detect and profile the interacting bioamphiphiles may give the opportunity to gain information about the health of animals much more rapidly and is potentially capable of used by lay technicians without the necessity of specialized equipment. Owing to the complexity of the gut environment within the zebrafish, another option for sensing could be to attach antibodies to the droplet interface, as has been previously explored with thin nematic LC films.^24^ This approach could maintain the stability of the droplets within the gut while still preserving the high sensitivity of the assay: for example, such specific droplets could provide rapid feedback about pathogenic conditions by detecting specific markers of disease. Research would be necessary, however, to investigate the mobility of macromolecules, such as proteins, across any protective coatings we use to enable the droplets’ survival.

In conclusion, we have demonstrated the potential of CLC droplets for biological sensing, both *in vitro* and *in vivo*. There is a wide scope in terms of what can be detected, automation in signal analysis, and into what novel platforms we can incorporate CLCs such as simpler fatty acid biosensors compared to those based on genetic modifications^68^ and enzymatic reactions.^69^ Future work can proceed in several directions: to test the limits of CLCs for sensing, both in terms of qualitative and quantitative analysis; the use of CLCs when incorporated into specific immunoassay sensors;^24^ to produce the droplets in a high-throughput, controlled (e.g., to obtain monodisperse samples) fashion using microfluidic systems;^70^ and to further store them more efficiently to improve detection.^64,65^

## Experimental Section

### Materials

The nonchiral nematic liquid crystal 4-cyano-4′-pentylbiphenyl (5CB, 95%+, Figure S1a) was obtained from Tokyo Chemical Industries. The chiral nematic liquid crystal mixture was prepared from the eutectic liquid crystal blend RO-TN 407 (F. Hoffmann-La Roche) mixed with 35% w/w chiral dopant CB15 ((*S*)-4-cyano-4′-(2-methylbutyl)biphenyl, Synthon GmbH, Figure S1b), producing a red-reflecting LC mixture (λ ∼ 650 nm), and was graciously provided by the University of Luxembourg. Poly(vinyl alcohol) (PVA) in two weights (high weight: *M*_w_ = 30–70 kDa, 87–89% hydrolyzed; low weight: *M*_w_ = 13–23 kDa, 87–89% hydrolyzed), sodium dodecyl sulfate (SDS, 99%+, Figure S1d), and dimethyloctadecyl[3-(trimethoxysilyl)propyl]ammonium chloride (DMOAP, 42% solution in methanol) were obtained from Merck–Sigma-Aldrich. Trizma base (tris(hydroxymethyl)amino-methane), and Trizma HCl (tris(hydroxymethyl)aminomethane hydrochloride) were sourced from Sigma-Aldrich and combined in ultrapure water to obtain a buffer with pH 7.4 (Tris 7.4). Lauric acid (dodecanoic acid, LA, 99%, Figure S1c) was obtained from Acros Organics. The phospholipids DOPC (1,2-dioleyoyl-*sn*-glycero-3-phosphocholine, 25 mg mL^–1^ in chloroform), Liss-Rhod DOPE (1,2-dioleyoyl-*sn*-glycero-3-phosphoethanolamine-N-(lissamine rhodamine B sulfonyl) (ammonium salt), Figure S1f, 1 mg mL^–1^ in chloroform), and DLPC (1,2-dilauroyl-*sn*-glycero-3-phospho-choline, Figure S1(e), 25 mg·ml^–1^ in chloroform) were sourced from Avanti Polar Lipids. Kdo2-Lipid A (di[3-deoxy-*D*-manno-octulosonyl]-lipid A (ammonium salt), Figure S1h, lyophilized powder, 90%+) was purchased from Merck–Sigma-Aldrich. Soy saponin extract (95% pure, Figure S1g, kindly provided by T. Kortner, NMBU Oslo, Norway; origin: Organic Technologies, Coshocton, OH, USA). Sodium butyrate powder (≥98.5%) used in zebrafish studies was obtained from Sigma-Aldrich. Ultrapure deionized water (resistivity 18.2 MΩ cm) was purified with a Milli-Q system.

### Solutions

We prepared stock solutions of 0.2% w/w PVA (of both weights), 6.0 and 12.0 mM SDS, and 5.0 mM LA in Tris 7.4 buffer and allowed them to mix at room temperature until the powders were fully dissolved, as verified by the presence of an optically transparent solution with no lumps or aggregates.

For phospholipids, appropriate volumes of lipids in chloroform were pipetted into a clean glass vial. Chloroform was then evaporated under vacuum until the films were dried, after which the lipid vials were hydrated with the buffer solution at room temperature for at least 4 h. Lipid dispersions were either then sonicated using a pulsed tip probe sonicator (10% power, 0.1 s on/0.9 s off) for 5 min or extruded twice through a 0.22 μm PTFE syringe filter into an Eppendorf tube to obtain a clear, optically transparent dispersion. In the case of Kdo2-Lipid A, we directly massed the appropriate quantity of powder into an Eppendorf tube before adding an appropriate quantity of buffer solution, agitating, and filtering to obtain a clear dispersion. All solutions were used within a week of preparation.

### Protocols

#### Droplet Visualization, Drying, and Rehydration

We visualized the LC droplets both in bulk solutions and when dried on glass slides. For bulk LC droplet samples, we pipetted 250–1000 μL of bath solution along with 2.5–5.0 μL LC solution in a clean 1.5 mL Eppendorf tube, in order to obtain a dilute dispersion of droplets. This dispersion was alternately vortex-mixed at a high power for 30 s and manually shaken to obtain a cloudy dispersion. We then immediately pipetted 10–20 μL of dispersion onto a clean glass slide or into a 5 mm wide polydimethylsiloxane (PDMS) well covalently bonded to a glass coverslip through plasma bonding.

To prepare microscopy slides with dried arrays of LC droplets, fresh glass slides were either simply rinsed with Milli-Q water and air-dried with a compressed air pistol or silanized by plasma cleaning using a Harrick PDC-32G plasma cleaner/sterilizer for 60 s followed by immediate immersion in a dilute solution of DMOAP (1% v/v DMOAP in ultrapure water) for 30 min, with gentle shaking throughout to ensure all glass surfaces were adequately exposed to the surfactant. The treated slides were then washed at least three times with deionized water before baking for 4 h to overnight at 115 °C in an oven. The success of the surface treatment was verified by a simple contact angle observation with ultrapure water to check for wetting.

Two to three droplets (10 μL each) of the prepared LC droplet dispersion were deposited on each glass slide (hydrophobized or untreated) with a minimum separation of 10 mm between each droplet. The slides were allowed to dry overnight under ambient conditions (20 °C, 55% RH). A 10 μL droplet of analyte solution was then gently pipetted on top of the dried droplet array and continuously imaged with manual focus adjustments during video acquisition. Hydrophobization of the glass slides appeared to improve the longevity and survivability of the droplets, but created issues with lensing when rehydrating the droplet arrays for sensing.

To capture images and videos, we used an Olympus BX60 polarizing optical microscope equipped with a DT70 color camera and operated with manufacturer-provided software for the reflection-mode and color micrographs and a Nikon Ti2 Eclipse microscope with a CoolLED light source for epifluorescence measurements.

#### Image Analysis

The color images of droplets in varied microenvironments were analyzed using MATLAB R2019b to estimate both the intensity values and the mutual ratios between RGB channels. The values of the three channels were defined as the average intensities of the respective channels for each droplet. One-way analysis of variance (ANOVA, α = 0.01) was used to confirm if there was any significant difference for the R/G, R/B, or G/B ratios between droplets treated by different amphiphiles. Error bars represent the standard error of the mean (*n* > 50 droplets for each sample). Response time was analyzed by selecting a minimum of 20 random droplets in a video and recording the timestamps at which switching began (typically indicated by a blue “flash”) to the time when a steady state was reached, the progression of which is illustrated in Figure 6a. This approach was additionally used for determining color ratios as a function of time, where we selected 10 random droplets and determined the color intensities and ratios at 20 s time intervals for 0.6 mM SDS and 10 s time intervals for 6.0 mM SDS solutions.

#### Surface Tension Measurements

Surface tension measurements were performed with a Sinterface PAT-1D pendant drop tensiometer with accompanying software. A stainless steel cannula (ID 1.94 mm) was flushed alternately with Milli-Q water and 96% w/w ethanol to clean the system prior to each measurement with a new material. A droplet with a constant surface area (20 mm^2^) was generated and allowed to equilibrate for at least 30 min, with the surface tension determined from the average calculated surface tensions once the droplet reached a steady state. Surface tensions were calculated using a drop-shape fitting technique similar to those described in literature.^71^ Each measurement was performed in duplicate to ensure consistency, with the final presented data point representing the average of the two measurements.

#### In Vivo Experiments

Wildtype (AB) zebrafish were grown both in either E2 buffer (5 mM NaCl, 0.17 mM KCl, 0.33 mM CaCl_2_, and 0.33 mM MgSO_4_), from days 2–5 postfertilization in a E2 buffer solution containing 0.5 mg·ml^–1^ soy saponin extract, or from days 3–5 in 5 ng/mL butyrate added to E2 buffer. Zebrafish grown in media containing soy saponin or butyrate were then washed with fresh E2 buffer to remove excess saponin or butyrate. Once they reached age 5 dpf, they were anaesthetized in 3-amino benzoic acid ethyl ester (tricaine/ethyl 3-aminobenzoate; Sigma-Aldrich; 168 μg mL^–1^ in Tris pH 7) and embedded in 1% low melting point agarose (UltraPure Agarose, ThermoFisher Scientific) in E2 medium containing a small amount of anesthetic (168 μg mL^–1^ Tricaine). Zebrafish were orally gavaged^72^ with 4 nL of the CLC mixture using hydrophobized glass capillaries (1.0 OD × 0.78 ID × 100 L mm, Harvard apparatus, treated with oxygen plasma and immersion in DMOAP solution to create hydrophobic glass) by means of a micromanipulator and Eppendorf FemtoJet setup. Zebrafish were imaged using a Leica DM6 upright microscope and an Olympus polarizing optical microscope equipped with a DT70 color camera.

## Supporting Information Available

Additional experimental results and parameters, including chemical structures (Figure S1), additional micrographs from *in vitro* and *in vivo* experiments (Figures S3, S7, S9, and S10), and statistical analyses of droplet size, color, switching time, and significance of differences (Figures S2, S4, S5, S6, and S8). (PDF)
Video S1 showing the dynamics of the switching of arrays of the CLC droplets with 0.6 mM SDS solution (MP4)
Video S2 showing the dynamics of the switching of arrays of the CLC droplets with 6.0 mM SDS solution (MP4)
Video S3 showing the dynamics of the switching of arrays of the CLC droplets with 12.0 mm SDS solution (MP4)
Video S4 showing the dynamics of the switching of arrays of the CLC droplets with 5.0 mM lauric acid solution (MP4)
