# Supplementary material for: Designing Biological Microsensors with Chiral Nematic Liquid Crystal Droplets
Source: ACS Appl Mater Interfaces. 2022 Aug 15;14(33):37316–29. doi: 10.1021/acsami.2c06923 (PMC9412956; doi:10.1021/acsami.2c06923)
Supplement: Supplementary file 1 — am2c06923_si_001.pdf [file am2c06923_si_001.pdf]

Supporting Information for "Designing Biological Micro-Sensors with Chiral Nematic Liquid Crystal Droplets"

Lawrence W. Honaker[a], Chang Chen[a], Floris M.H. Dautzenberg[a], Sylvia Brugman[b], and Siddharth Deshpande[a]

[a] *Physical Chemistry and Soft Matter, Wageningen University & Research, 6708 WE Wageningen, The Netherlands*

[b] *Host–Microbe Interactomics, Wageningen University & Research, 6708 WD Wageningen, The Netherlands*

Chemical Structures

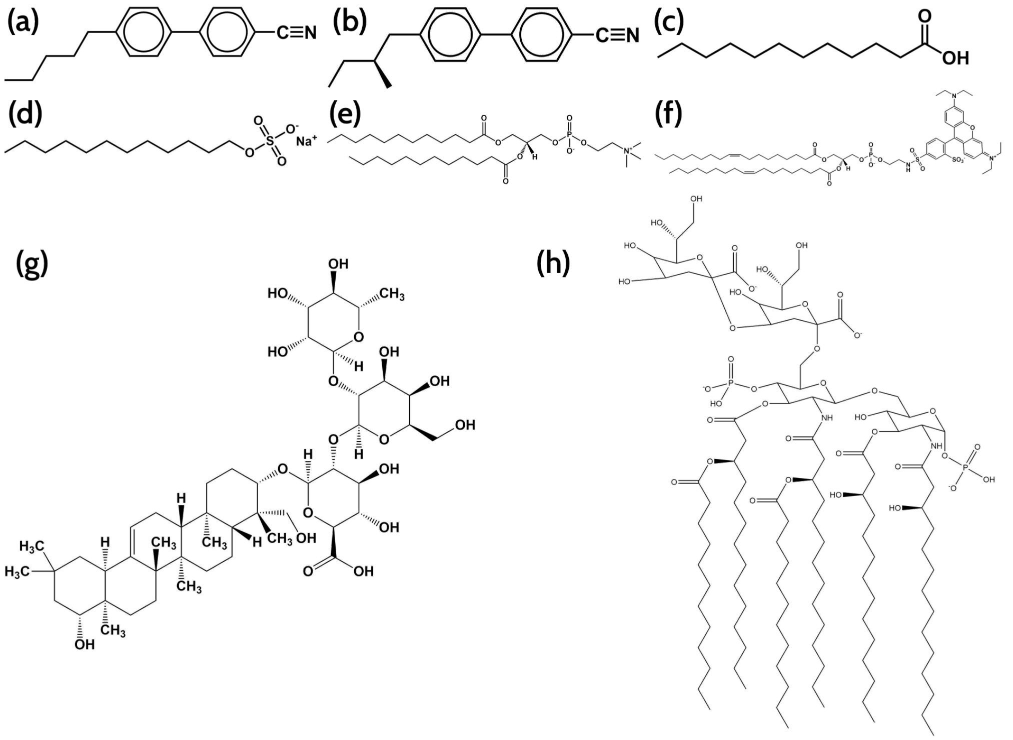

Figure S1: **Structures of the main chemical compounds (liquid crystals, amphiphiles, and proteins) used in this work.** (a) 4-cyano-4′-pentylbiphenyl (5CB); (b) (*S*)-4-cyano-4′-(2-methylbutyl)biphenyl (CB15); (c) dodecanoic/lauric acid (LA); (d) sodium dodecyl sulfate (SDS); (e) 1,2-dilauroyl-*sn*-glycero-3-phosphocholine (DLPC); (f) 1,2-dioleyoyl-*sn*-glycero-3-phosphoethanolamine-N-(lissamine rhodamine B sulfonyl) (ammonium salt) (Liss-Rhod DOPE); (g) soyasaponin; and (h) Kdo2-Lipid A (di[3-deoxy-*D*-manno-octulosonyl]-lipid A (ammonium salt)).

Microscopy and Supplemental Data

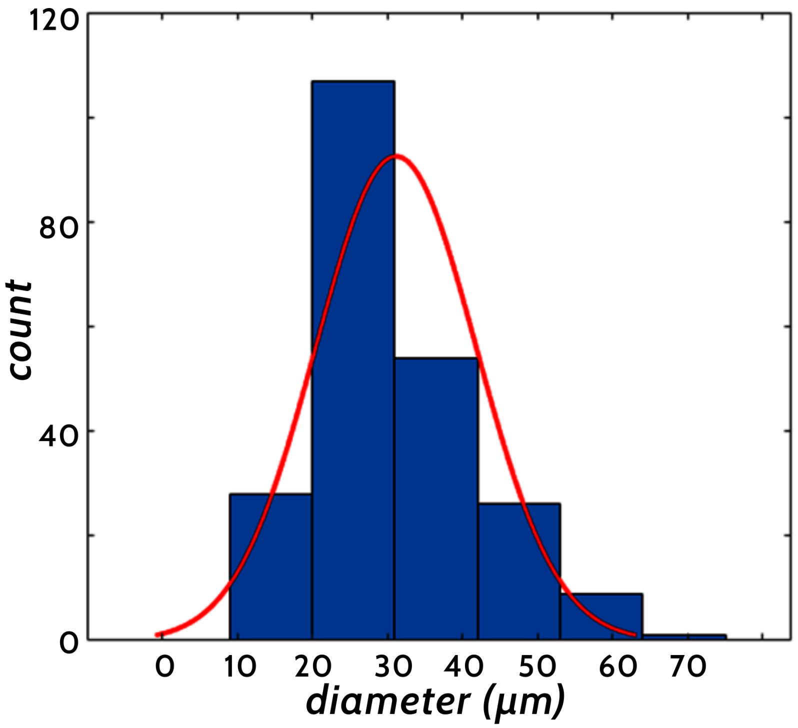

Figure S2: **Droplets analyzed during these experiments were generally uniform in size.** Histogram showing the combined size distribution of droplets produced in three different experiments with lauric acid as the sensing amphiphile, showing an average droplet size of 33±11 μm (mean ± standard deviation) across 264 droplets.

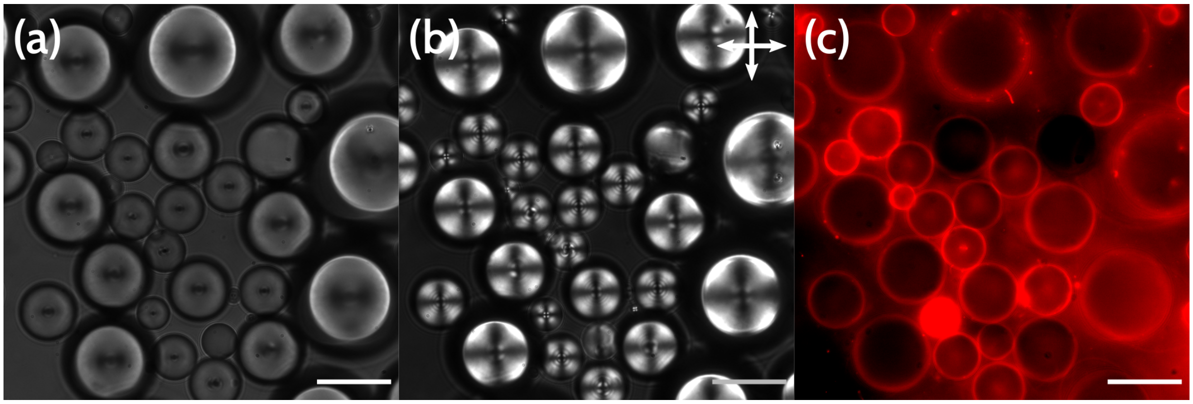

Figure S3: **Fluorescence microscopy shows us where and how lipids adsorb at the LC-water interface.** Droplets of 5CB prepared in a solution of 4 μM DOPC with 1:500 added Liss-Rhod DOPE in water, viewed (a) in transmission mode without polarizers; (b) between crossed polarizers, showing the characteristic Maltese cross textures of homeotropic-/normal-aligned NLC droplets; and (c) with epifluorescence, showing fluorescence signals corresponding to lipids adsorbed at the interface of the droplet. The adsorbed lipid is a proximal cause of the change of alignment. Scale bars 25 μm.

	PVA 0.2% w/w	SDS 0.1 mM	SDS 0.6 mM	SDS 6.0 mM	lauric acid 0.1 mM	lauric acid 1.0 mM	lauric acid 3.0 mM	lauric acid 5.0 mM	Kdo2-Lipid A 0.1 mM	Kdo2-Lipid A 1.0 mM
PVA 0.2% w/w		all	all	all	all	R/G R/B	R/G R/B	all	all	all
SDS 0.1 mM			all	R/B G/B	R/B	all	all	all	R/G R/B	R/G R/B
SDS 0.6 mM				G/B	all	G/B	all	R/G G/B	all	R/G R/B
SDS 6.0 mM					all	G/B	R/G G/B	all	all	all
lauric acid 0.1 mM						all	all	all	R/G R/B	R/G G/B
lauric acid 1.0 mM							none	none	all	R/G R/B
lauric acid 3.0 mM								none	all	R/G R/B
lauric acid 5.0 mM									all	all
Kdo2-Lipid A 0.1 mM										R/B G/B
Kdo2-Lipid A 1.0 mM										

Figure S4: **An analysis matrix showing that the ratios of the primary color intensities can be used to distinguish between different amphiphiles.** Each colored box (R/G in brown, R/B in magenta, and G/B in cyan) indicates a comparison for which $p \leq 0.001$ when comparing droplets prepared from 0.2% PVA; 0.1, 0.6, and 6.0 mM SDS; 0.1, 1.0, 3.0, and 5.0 mM lauric acid; and 0.1 and 1.0 mM lipid A (CMC unknown). As can be seen, at least one color ratio was statistically significant for almost all samples at the chosen significance interval.

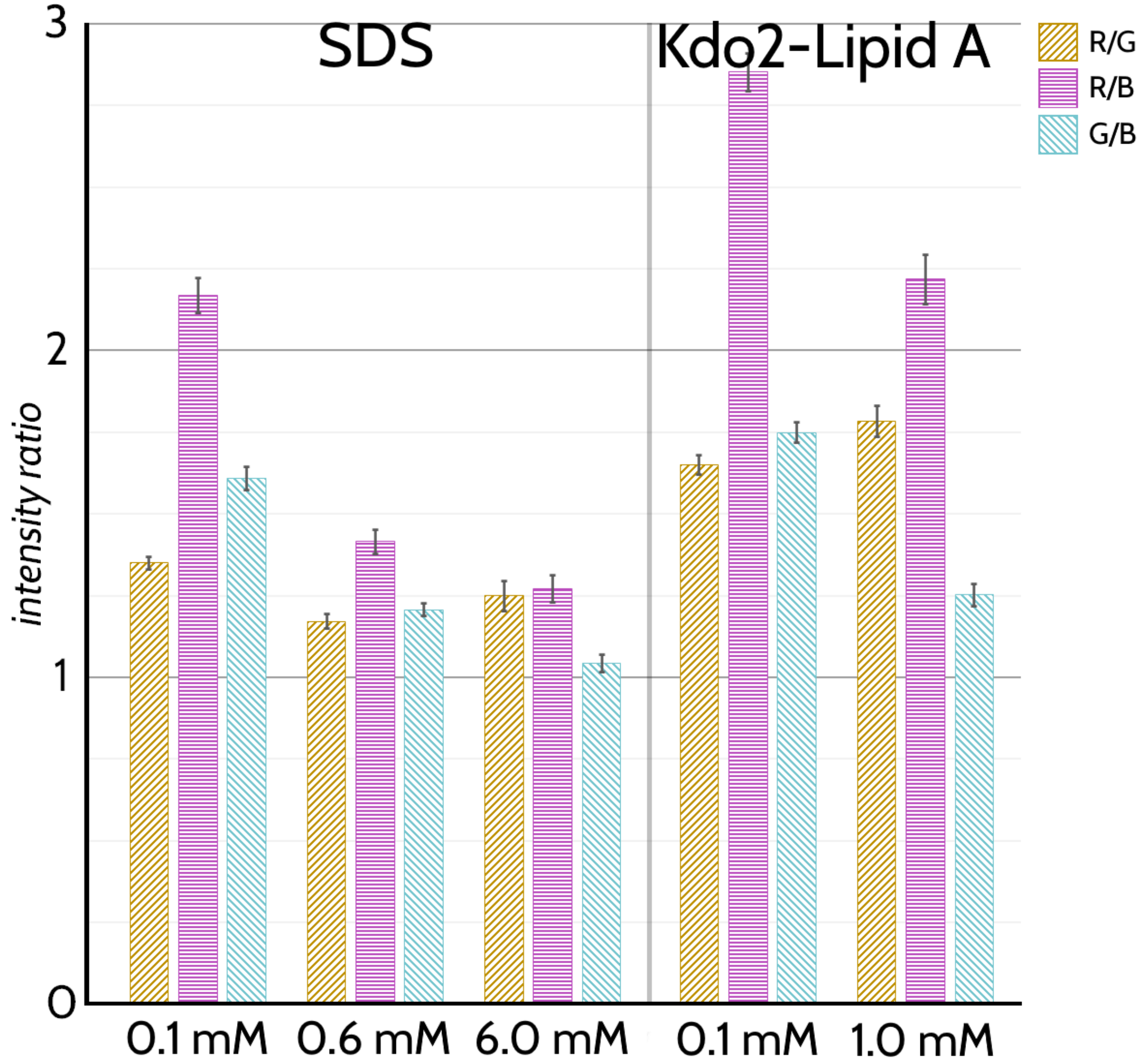

Figure S5: **The use of the ratios of color channels can act as amphiphile-specific signatures.** Normalized R/G, G/B, and B/G ratios for CLC droplets exposed to varied concentrations in bulk samples of each of the amphiphiles not previously presented (here, SDS and Kdo2-Lipid A). At least 50 droplets were measured for each group. Error bars indicate the calculated standard error of the mean.

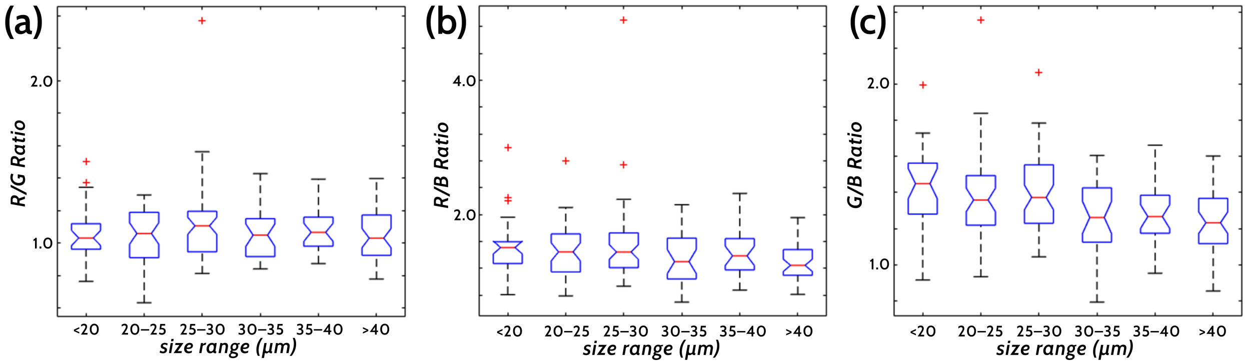

Figure S6: **The analysis of droplet color intensity is largely size independent.** (a-c) Box-and-whisker plots showing the effects of the size distribution on the (a) R/G; (b) R/B; and (c) G/B channel ratios in case of lauric acid. The central line indicates the median, with the boxes denoting the second and third quartiles of the data and the whiskers indicating the interquartile range, with red dots indicating the outliers of the interquartile range. We saw no effects of size on the channel intensities, showing the final colors were largely droplet size-independent.

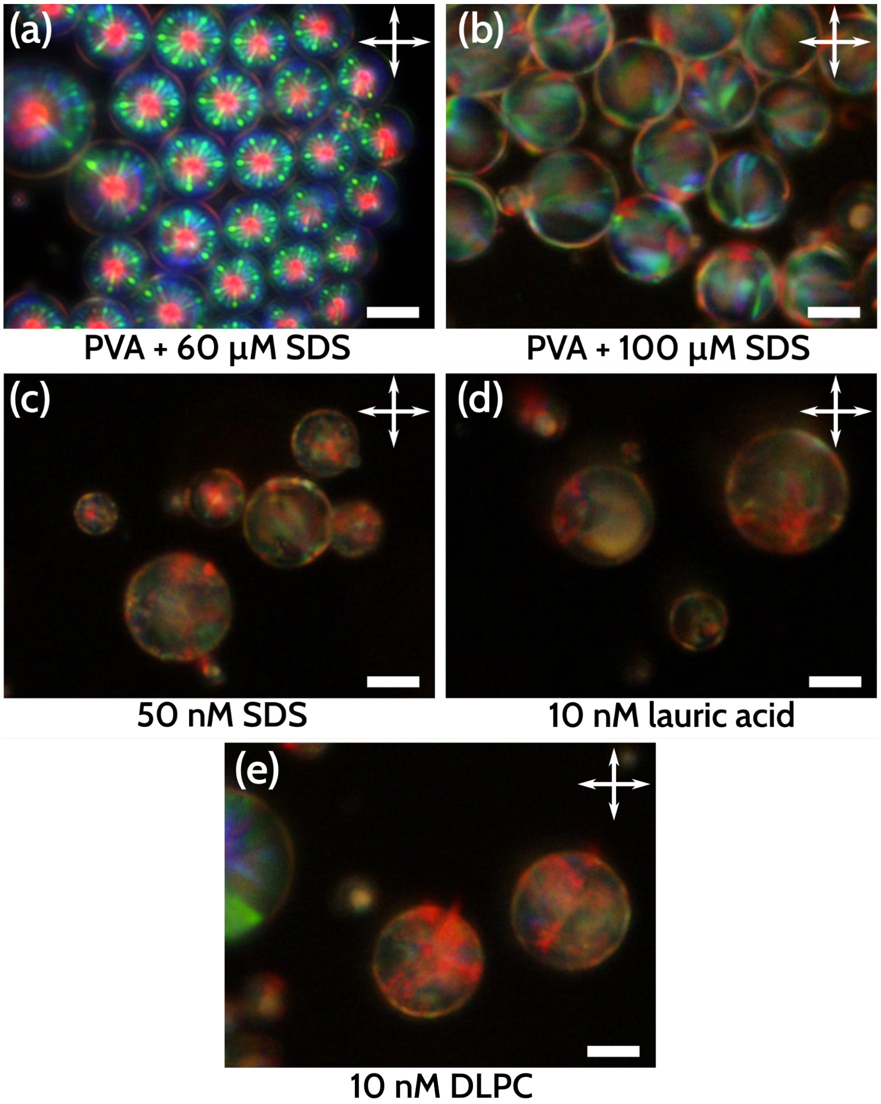

Figure S7: **The presence of PVA affects the threshold sensitivity of switching for LC droplets.** POM micrographs of a CLC sample (a-b) prepared in 0.2% PVA, dried, and rehydrated (a) with 60 μM SDS solution, showing no difference from a sample containing just PVA (Figure 2(a)), and (b) with 100 μM SDS solution, showing a response in the droplets. By comparison, droplets of the CLC prepared in (c) prepared in 50 nM SDS solution without PVA, (d) in 10 nM LA solution, and (e) in 10 nM DLPC solution showed clear switching. While lower amphiphile concentrations could cause switching, droplets became difficult to suspend in solution at those concentrations. The presence of PVA coating the droplets greatly decreases the sensitivity of the microdroplets to amphiphiles that they ordinarily would sense at nanomolar concentrations. Scale bars 25 μm.

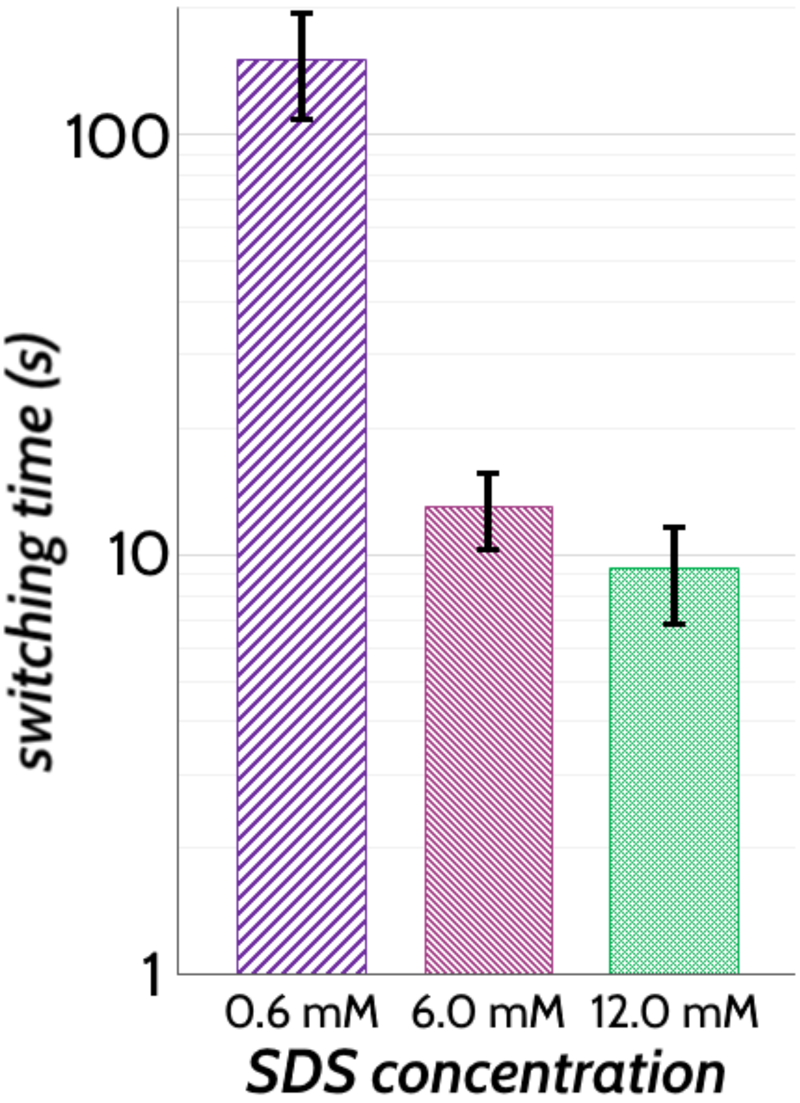

Figure S8: **CLC droplets dried in arrays can switch quickly, dependent on the concentration of amphiphile present.** By exposing a dried array of droplets to different concentrations of an amphiphile (here, SDS), we observe marked differences in the switching times, ranging from < 10 s for 12.0 mM SDS solution to > 2 min for 0.6 mM SDS solution. Analysis based on the switching times of at least 20 randomly selected droplets in each batch. Error bars indicate the standard deviation of the mean.

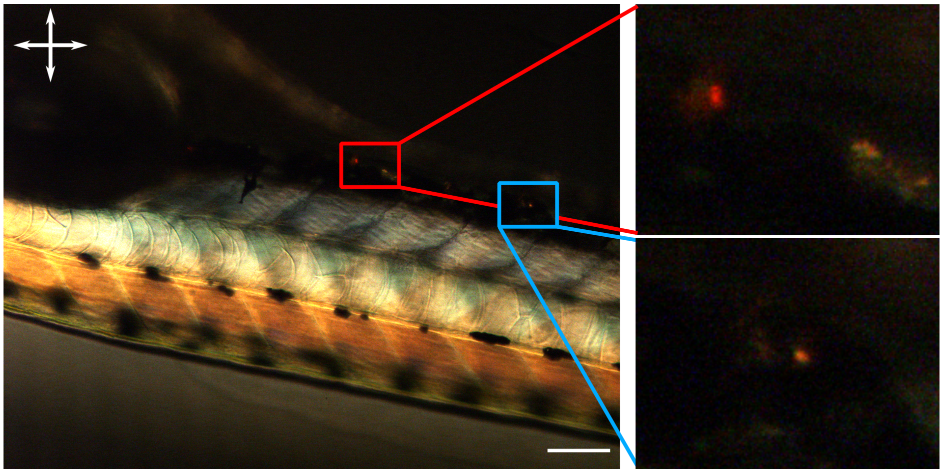

Figure S9: **Zebrafish melanocytes and bones, while birefringent, do not show the same optical signature as CLC droplets.** A POM micrograph of a zebrafish, viewed between crossed polarizers, showing the birefringence of both the cartilage/bones and the melanocytes (insets). Scale bar 25 μm.

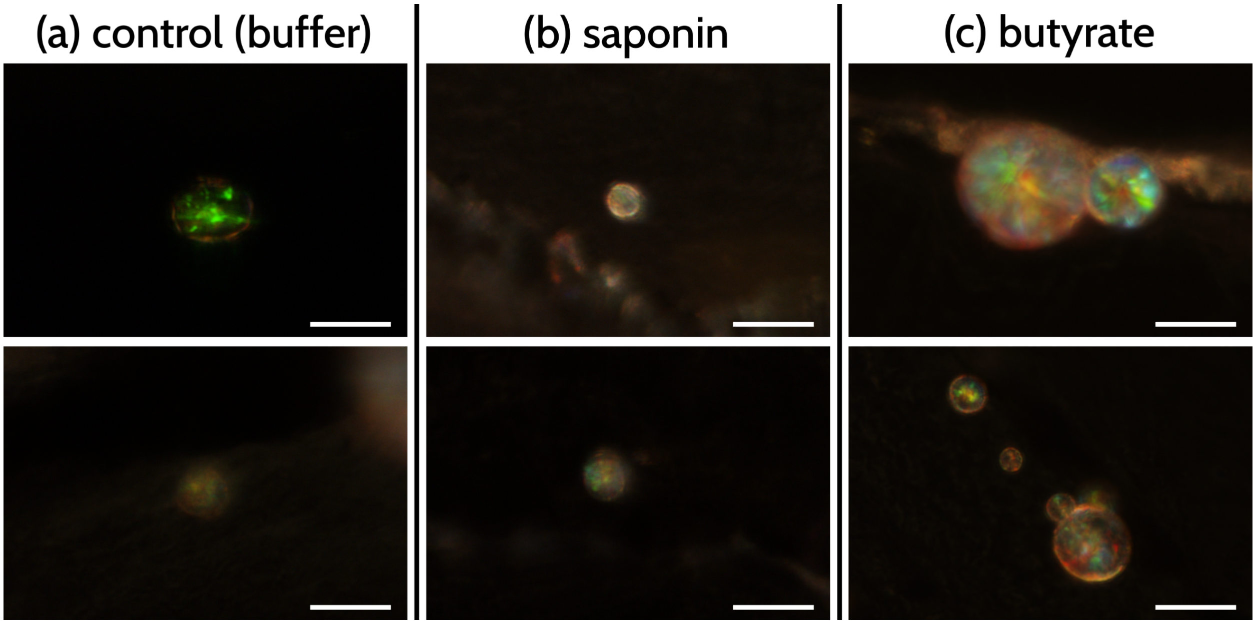

Figure S10: **CLC droplets subjected to different gut environments can show different optical responses.** Droplets of our red-reflecting CLC mixture were prepared in PVA buffer solution. We then gavaged 5 dpf zebrafish larvae (a) grown in E2 buffer (without any inflammatory agents), acting as a negative control, and (b) subjected to soy saponin, which causes intestinal inflammation, and (c) butyrate, a short-chain fatty acid product that can be indicative of intestinal damage. Scale bars 50 μm. Images taken between crossed linear polarizers.

Video Captions

Video S1: Rehydration of a CLC droplet array, suspended in 0.2% PVA solution and dried on a glass slide, with 0.6 mM SDS solution. Viewed in reflection mode between crossed linear polarizers. Video accelerated 20×.

Video S2: Rehydration of a CLC droplet array, suspended in 0.2% PVA solution and dried on a glass slide, with 6.0 mM SDS solution. Viewed in reflection mode between crossed linear polarizers. Video accelerated 5×.

Video S3: Rehydration of a CLC droplet array, suspended in 0.2% PVA solution and dried on a glass slide, with 12.0 mM SDS solution. Viewed in reflection mode between crossed linear polarizers. Video accelerated 5×.

Video S4: Rehydration of a CLC droplet array, suspended in 0.2% PVA solution and dried on a glass slide, with 5.0 mM lauric acid solution. Viewed in reflection mode between crossed linear polarizers. Video accelerated 5×.
